# Seaweed Nutritional Value and Bioactive Properties: Insights from *Ascophyllum nodosum*, *Palmaria palmata*, and *Chondrus crispus*

**DOI:** 10.3390/life14111522

**Published:** 2024-11-20

**Authors:** Natália Čmiková, Przemysław Łukasz Kowalczewski, Dominik Kmiecik, Aneta Tomczak, Agnieszka Drożdżyńska, Mariusz Ślachciński, Łukasz Szala, Sanja Matić, Tijana Marković, Suzana Popović, Dejan Baskic, Miroslava Kačániová

**Affiliations:** 1 Institute of Horticulture, Faculty of Horticulture and Landscape Engineering, Slovak University of Agriculture, Tr. A. Hlinku 2, 94976 Nitra, Slovakia; 2 Department of Food Technology of Plant Origin, Poznań University of Life Sciences, 31 Wojska Polskiego St., 60-624 Poznań, Poland; przemyslaw.kowalczewski@up.poznan.pl (P.Ł.K.); dominik.kmiecik@up.poznan.pl (D.K.); 3 Department of Biochemistry and Food Analysis, Poznań University of Life Sciences, 48 Mazowiecka St., 60-623 Poznań, Poland; aneta.tomczak@up.poznan.pl; 4 Department of Biotechnology and Food Microbiology, Poznań University of Life Sciences, 48 Wojska Polskiego St., 60-627 Poznań, Poland; agnieszka.drozdzynska@up.poznan.pl; 5 Institute of Chemistry and Technical Electrochemistry, Poznań University of Technology, 4 Berdychowo St., 60-965 Poznań, Poland; mariusz.slachcinski@put.poznan.pl; 6 Students’ Scientific Club of Food Technologists, Poznań University of Life Sciences, 31 Wojska Polskiego St., 60-624 Poznań, Poland; szala.lukasz99@gmail.com; 7 Department of Pharmacy, Faculty of Medical Sciences, University of Kragujevac, Svetozara Markovića, 69, 34000 Kragujevac, Serbia; sanjad.matic@gmail.com (S.M.); tianafeels@gmail.com (T.M.); 8 Centre for Molecular Medicine and Stem Cell Research, Faculty of Medical Sciences, University of Kragujevac, Svetozara Markovića, 69, 34000 Kragujevac, Serbia; popovic007@yahoo.com (S.P.); dejan.baskic@gmail.com (D.B.); 9 School of Medical and Health Sciences, University of Economics and Human Sciences in Warsaw, Okopowa 59, 01043 Warszawa, Poland

**Keywords:** macroalgae, protein content, fatty acids, antioxidant activity, cytotoxic activity

## Abstract

This study investigates the nutritional composition and bioactive properties of *Palmaria palmata* (dulse), *Ascophyllum nodosum* (knotted wrack), and *Chondrus crispus* (Irish moss). Understanding the nutritional values of these seaweeds is very important due to their potential health benefits, especially their antioxidant properties and cytotoxic activities, which point to their ability to inhibit cancer cell proliferation. Comprehensive analyses were conducted to assess protein content, amino acid composition, mineral profile, fatty acids, polyphenols, total carotenoids, antioxidant activity, and cytotoxicity against cervical (HeLa), and colon (HCT-116) cell lines. *P. palmata* exhibited the highest protein content, while *C. crispus* was richest in calcium, iron, manganese, and zinc. Amino acid analysis revealed *C. crispus* as being particularly high in essential and non-essential amino acids, including alanine, glutamic acid, and glycine. *A. nodosum* and *C. crispus* were rich in polyunsaturated fatty acids (PUFAs), notably eicosapentaenoic acid (EPA), and docosahexaenoic acid (DHA). *A. nodosum* showed the highest total carotenoid content. Polyphenol analysis highlighted the presence of compounds such as *p*-coumaric acid, gallic acid, and *p*-hydroxybenzoic acid across the species. Both the ethanolic and hexane *A. nodosum* extracts demonstrated the strongest antioxidant potential in DPPH^•^ and ABTS^+^ assays. The cytotoxicity evaluation revealed high anticancer activity of *A. nodosum* and *C. crispus* hexane extract against HeLa and HCT-116, though it employed cell cycle arrest and apoptosis. *A. nodosum* hexane extract exhibited moderate selective anticancer activity against HCT-116. These findings underscore the nutritional diversity and potential health benefits of these macroalgae (seaweed) species, suggesting their suitability as functional foods or supplements, offering diverse nutritional and therapeutic benefits.

## 1. Introduction

Seaweeds (macroalgae) have long been integrated into both human and animal diets, with their protein compositions varying by species and seasonal fluctuations [1]. They hold significant cultural and culinary importance across various Asian regions, alongside numerous claimed health benefits. Research underscores their nutritional richness and health-promoting properties, distinguishing them from terrestrial plants and animal-derived foods. Seaweeds are packed with essential nutrients such as n3 fatty acids, essential amino acids, and a spectrum of vitamins including A, B, C, and E, uniquely positioned to support human well-being These marine plants also harbor a diverse array of minerals and non-nutrient components, such as fiber and polyphenols, further enhancing their nutritional profile and health benefits [2]. Epidemiological studies worldwide suggest a notable trend: countries where seaweed is a dietary staple often exhibit lower rates of obesity and diet-related diseases [3]. Due to their low-calorie content and dense concentration of fiber, minerals, and vitamins, seaweeds have emerged as promising sources of nutritious and healthful foods [4]. Moreover, seaweeds exhibit cytotoxic effects that contribute to their potential therapeutic applications [5].

As the demand grows for sustainable protein sources, seaweed proteins show promise across pharmaceutical, nutraceutical, cosmetic, food, and feed industries. Certain species boast significant protein content, rivaling traditional plant proteins in their unique amino acid composition [6]. The protein content of seaweed can range up to 50% of its dry weight, influenced by factors like growth conditions, harvesting time, and seaweed species [4]. Fleurence et al. [7] have noted that the protein content is usually higher in red seaweed species (from 20% to 47% of dry weight), compared with green species, which have protein contents of roughly 9% to 26% of dry weight. Brown seaweeds usually have the lowest protein content compared with red and green seaweeds, ranging from 3% to 15% of dry matter. The essential amino acid compositions of certain seaweed species, such as *Porphyra* sp. (Rhodophyta), have been shown to be comparable to those found in soy and egg protein sources. Moreover, many seaweed species exhibit elevated levels of amino acids like arginine, aspartic acid, and glutamic acid [7,8,9]. All three groups of seaweeds (green, brown and red) have comparable amounts of amino acids. Essential amino acids were present in all seaweed species tested, with red seaweed species in particular showing higher concentrations of taurine compared with brown species [8].

Seaweeds are renowned for their ability to accumulate essential minerals and trace elements, varying by type and environment. Chlorophyta accumulates Mg and Fe, Rhodophyta contains higher Mn, and Phaeophyceae has elevated iodine levels. Red and brown seaweeds generally have more Na, K, and Zn compared with green seaweeds [10]. In a study of Bulgarian Black Sea coast seaweeds, green algae showed high levels of Mg, Na, Cr, Co, Fe, Mn, As, Pb, and Hg. Red algae were found to be rich in Cu, Zn, and Se, while brown algae had elevated Ni, Cd, Ca, K, Sr, and Ba. This rich mineral content makes seaweeds valuable for developing functional foods and as dietary supplements [11].

Seaweed are renowned for their abundant polyunsaturated fatty acids (PUFAs), which are essential nutrients crucial for human health [12]. Marine algae, despite their low lipid content, are recognized as a significant source of various bioactive nutritional compounds essential for a balanced diet. These PUFAs, including linoleic acid, arachidonic acid, and eicosapentaenoic acid (EPA), are particularly enriched in red and brown algae, which are noted for their high concentrations of omega-3 (n3) fatty acids [13]. Seaweeds are acknowledged also for their significant content of polyphenols, with a particular emphasis on phlorotannins. These polyphenolic compounds have attracted interest due to their wide range of biological effects, including antioxidant, antiproliferative, and antidiabetic properties [14]. Present in abundant quantities in green, red, and brown seaweed, polyphenols play a crucial role in enhancing their bioactive capabilities. Analyzing the polyphenol composition of seaweed involves comprehending their structural diversity and biological functions, which can vary depending on species and environmental factors [15].

Understanding the cytotoxic and antioxidant activity of seaweeds is very important as these properties directly contribute to their health effects and potential therapeutic applications. The rich nutritional composition of seaweeds, which includes essential amino acids, PUFAs, vitamins and minerals, provides the basis for their bioactive properties [16]. The antioxidants present in seaweed help to combat oxidative stress by neutralizing free radicals that can otherwise damage cells and lead to various diseases, including cancer. This antioxidant activity is closely linked to the presence of polyphenols, phlorotannins and other bioactive compounds in seaweed [17]. In addition, the cytotoxic activity of seaweed extracts against cancer cells highlights their potential as natural anticancer agents. This activity is often attributed to specific compounds such as polyphenols, sulphated polysaccharides and certain proteins and peptides that can induce apoptosis in cancer cells. These bioactive compounds not only contribute to the cytotoxic effects but also improve the overall nutritional profile of seaweeds, making them valuable for both dietary and therapeutic purposes [5,18,19]. Seaweed antioxidants are becoming more acknowledged for their capability to combat oxidative stress and associated health concerns. These compounds encompass polysaccharides, phenolics, phycobiliproteins, and PUFAs, which counteract free radicals and shield against lipid damage and reactive oxygen species (ROS). Their efficacy depends on their molecular structure and biochemical characteristics. Seaweed is increasingly viewed as a natural substitute for synthetic antioxidants in pharmaceuticals, functional foods, and supplements. This overview emphasizes seaweed antioxidants, emphasizing their mechanisms and health advantages [20,21,22].

*Palmaria palmata*, widely known as dulse, is a prominent and valued red macroalga found along the North Atlantic coast [23]. These algae can reach lengths of up to 50 cm, though they typically grow to around 10 cm [24]. This commercially significant species has a long history of use as a food source, with records dating back centuries [24]. *P. palmata* is rich in carbohydrates (up to 66% dry weight) [25]. Its protein content, reaching up to 35% dry weight, includes essential amino acids, making it a beneficial protein source, particularly in winter. Additionally, it contains high levels of EPA, a beneficial fatty acid, and essential minerals like iodine, calcium, and magnesium, though its mineral profile varies seasonally. While generally low in potentially toxic elements, regular monitoring is advised for food safety [24].

*Ascophyllum nodosum* or (kelp, knotted wrack) is a brown algae species typically found in the tidal waters of the North Atlantic Ocean [26]. *A. nodosum* features a diverse chemical composition, including major minerals like sodium, magnesium, potassium, chloride, and sulfate, with selective accumulation of potassium and sulfate. It serves as a biomonitor for environmental contaminants due to its ability to accumulate metals and radionuclides. The key organic compounds include alginic acid, lipids, mannitol, proteins, laminarans, and fucoidans, which have applications in food and pharmaceuticals [27].

*Chondrus crispus*, commonly known as Irish moss, is a widely consumed red seaweed typically found along rocky coastlines in the North Atlantic [28]. *C. crispus* has a diverse chemical composition, comprising 14.4% to 27% protein, 19.2% to 28% neutral sugars, and 9.4% to 17% sulfated sugars. This alga is rich in carbohydrates, proteins, and lipids, with notable mineral content, including high levels of sodium, potassium, calcium, and magnesium. Methanolic extracts contain flavonoids, tannins, and phenolic compounds, such as catechin and p-coumaric acid. Overall, *C. crispus* is a valuable source of bioactive compounds with applications in food and health industries [29].

In this study, we focused on three species of commercially sourced seaweed: *Palmaria palmata* (dulse), *Ascophyllum nodosum* (knotted wrack), and *Chondrus crispus* (Irish moss). These seaweeds were cultivated under controlled conditions to maintain their consistently high quality. The seaweeds and their extracts were analyzed for total protein content, amino acid profiles, mineral composition, and fatty acid contents. The aim of this research is to extend the current knowledge on the nutritional composition and health benefits of these seaweeds, focusing particularly on their antioxidant and cytotoxic activity. Research on the cytotoxic and antioxidant activity of seaweeds highlights the significant health benefits of these marine organisms. By elucidating the link between their rich nutritional content and bioactive properties, this study aims to improve our understanding of how seaweeds can be used to promote human health and combat disease. While significant research has been conducted on the nutritional and bioactive properties of *Palmaria palmata*, *Ascophyllum nodosum*, and *Chondrus crispus*, notable gaps remain in the literature. Comprehensive analyses comparing their effects on multiple health-related outcomes are limited. This study addresses this gap by evaluating the nutritional profiles and antioxidant and cytotoxic activities of extracts from these three seaweed species against cancer cell lines, utilizing ethanol and hexanol as solvents to compare their bioactivity.

## 2. Materials and Methods

### 2.1. Samples

In this study, powdered forms of 100% *Palmaria palmata* (dulse), *Ascophyllum nodosum* (knotted wrack), and *Chondrus crispus* (Irish moss) from controlled organic cultivation (DE-ÖKO-005) were used. The *P. palmata* powder originated from Ireland, while both the *A. nodosum* and *C. crispus* powders originated from Canada. The particle size of the powdered seaweed was approximately 0.25 to 0.5 mm. All samples were purchased from Biotiva, a German company (Straßlach-Dingharting, Germany), in 2022. They adhered to the EC Organic Regulation standards and were stored hermetically sealed in a dark, dry environment at room temperature (~4 °C).

### 2.2. Protein Content and Amino Acid Composition

The determination of protein content was conducted following the guidelines outlined in ISO 20483:2013 [30], employing the Kjeldahl method, using a nitrogen-to-protein conversion factor of 6.25.

In case of amino acid analysis, protein hydrolysis was carried out using two distinct methods: acidic and oxidative. Acidic hydrolysis, performed at 110 °C for 23 h, is effective at breaking down proteins into their constituent amino acids, including a wide range such as alanine, arginine, aspartic acid, glutamic acid, leucine, lysine, serine, threonine, tyrosine, valine, histidine, isoleucine, phenylalanine, proline, and glycine. On the other hand, oxidative hydrolysis, which involves a two-step process conducted at 4 °C for 16 h followed by 100 °C for 2 h according to method 994.12 [31], is specifically targeted at sulfur-containing amino acids like methionine and cystine. After the hydrolysis process, the determination of amino acid content followed the procedure outlined by Tomczak et al. [32]. This involved dilution and derivatization of the amino acids using AccQ^•^Tag reagents, in adherence to the manufacturer’s guidelines, subsequent to the evaporation of the samples. For the analysis, ultra-efficient liquid chromatography (UPLC) was utilized with a Shimadzu Nexera 2.0 system, incorporating various components, such as a binary solvent manager, autosampler, column heater, and PDA detector. Separation was achieved using an AccQ-Tag Ultra C18 column with the following specific conditions: a mobile phase flow rate of 0.6 mL/min and a column temperature of 55 °C. A gradient mixing 5% and 100% AccQ^•^Tag Ultra solvent was employed for separation, with detection occurring at 260 nm via the use of a PDA detector. Quantification was carried out using standards containing known concentrations of amino acids, which were then diluted and derivatized with borate buffer and AccQ^•^Tag reagents. The analysis was repeated multiple times to establish a calibration curve, enabling accurate determination of amino acid content. Finally, the amino acid content was expressed as grams per 16 g of nitrogen, which is equivalent to grams per 100 g of protein.

### 2.3. Determination of Mineral Profile

An innovative system employing high-pressure and high-temperature conditions, coupled with concentrated microwave energy, was utilized for the mineralization of algae powders. A detailed description of sample preparation and analysis validation has been provided earlier [33]. Briefly, the samples were placed in sealed vessels with a capacity of 30 mL, constructed from chemically modified Teflon (Hostaflon TFM). Subsequently, 3 mL of 60% nitric acid and 1 mL of 30% hydrogen peroxide were added to these vessels. Encased within a steel jacket, the assembly underwent a 10 min mineralization process facilitated by microwave energy, delivered by an antenna with a power output of 200 W. Following mineralization, the samples were diluted to a final volume of 25 mL.

For elemental analysis utilizing the ICP OES technique, an emission spectrometer featuring an inductively coupled plasma excitation source (IRIS HR, Thermo Jarell Ash, Kenilworth, NJ, USA) was employed. Quantitative analysis was performed using the calibration curve method. The elemental contents of calcium (Ca), magnesium (Mg), potassium (K), sodium (Na), copper (Cu), iron (Fe), manganese (Mn), zinc (Zn), and lead (Pb) were determined. Results are expressed in milligrams per gram of dry matter (mg/g DM) based on six independent readings, comprising three biological and two technical replicates.

### 2.4. Fatty Acids Composition

Extraction of fatty acids adhered to the well-established protocol elucidated by Folch et al. [34], meticulously following their detailed guidelines. Subsequent determination of fatty acid composition ensued, adhering strictly to the official AOCS Ce 1 h-05 method [35], with specific parameters extracted from previous literature [36]. For analysis, an Agilent 7820A gas chromatograph (Agilent Technologies, Santa Clara, CA, USA) was employed, featuring a flame ionization detector (FID) and an SLB-IL111 capillary column (100 m in length, 0.25 mm inner diameter, and 0.20 μm film thickness; Supelco, Bellefonte, PA, USA). The GC conditions were as follows: the oven temperature ranged from 150 °C to 200 °C increasing at 1.5 °C/min; the temperature of the injector and detector were 250 °C, the carrier gas was helium at 1 mL/min; the GC operated in split mode 1:10. The obtained results are expressed as percentages relative to the total fatty acid content, providing crucial insights into the lipid profile of the samples under investigation.

### 2.5. Total Carotenoids

One gram of the powdered algae sample was homogenized in a mortar and repeatedly washed with 10 mL of acetone until the sample reached a colorless state. The resulting extract was then filtered through filter paper (Whatman^®^ Grade 2 Sigma Aldrich, Darmstadt, Germany) and used for the determination of total carotenoid content. Petroleum ether was carefully introduced into a separatory funnel equipped with a Teflon stopcock. The acetone extract of the sample and distilled water were then added, allowing them to flow down the walls of the funnel. The resulting mixture underwent phase separation, and the aqueous phase was discarded. The petroleum ether phase was washed twice with distilled water to remove any remaining acetone. The petroleum ether phase was then collected in a volumetric flask and passed through a small funnel containing 0.5 g of anhydrous sodium sulfate to remove any remaining water. The volumetric flask was adjusted to the desired volume with petroleum ether, and the total carotenoid content was determined based on the molar absorption coefficient of *β*-carotene. The carotenoid concentration (mg/g) was calculated using the following formula [37]:

(1) $$TTC\left(mg/g\right)=\frac{A.r.V.10}{E.n}$$

where A is the absorbance at 445 nm, r is the sample dilution, V is the volume of the petroleum, E is the molar absorption coefficient (E^1%^_1 cm_ = 2620), n is the weight of the sample, and TCC represents the total carotenoid content.

### 2.6. Polyphenols Profile Composition

Polyphenolic compound analysis via high-performance liquid chromatography (HPLC) was conducted using an Agilent 1260 Infinity II liquid chromatograph (Agilent Technologies, Inc., Santa Clara, CA, USA), following the method detailed by Drożdżyńska et al. [38], using methanol extract (80% *v*/*v*) obtained according to the methodology described earlier [39]. The instrumentation comprised an autosampler (G7129A), a pump (G7111A), and a diode detector (G7115A) with a spectral range spanning from 190 to 400 nm. Phenolic compound separation was accomplished using an SB-C18 column (50 mm × 4.6 mm with 1.8 µm particle diameter, Agilent) maintained at a temperature of 25 °C. Elution utilized solvents A (water and acetic acid, 98:2 by volume) and B (methanol and acetic acid, 98:2 by volume) with the following gradient profile: 0 min at 2% B, 22 min at 40% B, 26 min maintained at 40% B, 28 min at 100% B, and finally returning to 2% B at 36 min. The flow rate was set at 0.75 mL/min, and a sample volume of 5 µL was injected onto the column. Quantitative calculations were performed based on peak areas using OpenLab CDS (Agilent Technologies, Inc., Santa Clara, CA, USA). Results are expressed as micrograms per gram of dry matter (µg/g DM), providing valuable insights into the polyphenolic composition of the samples.

### 2.7. Bioactive Properties of Seaweed Extracts

#### 2.7.1. Preparation of Extracts for Antioxidant and Cytotoxic Activity

Solvents (denatured ethanol, denatured hexane) were used for extraction of seaweed powder. The algae samples were weighed 50 g into a 1 L glass bottle, where 500 L of the solvent was then added. The samples were left in a shaking incubator (GFL 3031 in Burgwedel, Germany) for 24 h in the dark at 25 °C. The liquid component was filtered through filter paper (Whatman^®^ Grade 2, Dassel, Germany) and the algae powder was again covered with 500 mL of solvent and incubated on the shaker for another 24 h. This procedure was repeated 3 times. Extracts at 100% concentration were prepared using a vacuum rotary evaporator (Witeg Labortechnik, Wertheim, Germany). Evaporation was carried out in a water bath at 50 °C and pressures of 97 mBar (for ethanol) and 264 mBar (for hexane). The extracts were transferred from the flask into closable glass containers using a metal spoon. The extract thus prepared was stored in the dark in a refrigerator at (±4 °C) and prepared for their analyses. The concentrated extracts were diluted to the given concentrations before antioxidant and cytotoxic activity. The extracts were dissolved into 99.5% dimethyl sulfoxide (DMSO) (Sigma Aldrich, St. Louis, MO, USA). The extract with DMSO was placed in an ultrasonic bath (Kraft & Dele, Łódź, Poland) at 30 °C for 30 min and then the extract was vortexed. This process was repeated until the extract was completely dissolved in DMSO.

#### 2.7.2. Antioxidant Activity

The effectiveness of ethanolic and hexane algae extracts as antioxidants was assessed using radical scavenging assays with two compounds: 2,2-diphenyl-1-picrylhydrazyl (DPPH^•^) and 2,2′-azino-bis(3-ethylbenzothiazoline-6-sulfonic acid) (ABTS^+^), both acquired from Sigma Aldrich (Taufkirchen, Germany). The extracts (ethanol and hexane) were dissolved in 100% DMSO to a concentration of 50 mg/mL. DPPH^•^ was dissolved in methanol at a concentration of 0.025 g/L and its absorbance adjusted to 0.8 at 515 nm using a Glomax spectrophotometer from Promega Inc. (Madison, WI, USA). The ABTS^+^ radical cation was prepared according to a previously outlined method and diluted to an absorbance of 0.7 at 744 nm prior to analysis [40]. In the assay protocol, 190 μL of either the DPPH^•^ or ABTS^+^ solution was combined with 10 μL of the algae extracts in a 96-well microtiter plate. This mixture was then incubated for 30 min at room temperature in the dark, with continuous shaking at 1000 rpm. Subsequently, absorbance reductions were measured at 744 nm and 515 nm for the ABTS^+^ and DPPH^•^ assays, respectively. The percentage inhibition of DPPH^•^ or ABTS^+^ was computed using the following formula:

(2) $$AA=\frac{\left(A_{0-A_{A}}\right)}{A_{0}}\times 100$$

where AA is antioxidant activity, A_0_ represents the absorbance of DPPH^•^ or ABTS^+^ with methanol, and A_A_ represents the absorbance of the sample. To ascertain the total antioxidant capacity, Trolox^®^ was dissolved in methanol (Uvasol^®^ for spectroscopy, Merck, Darmstadt, Germany) over a concentration range of 0–100 µg/mL and utilized as a standard reference. The findings are expressed in accordance with the Trolox^®^ calibration curve (TEAC). This meticulous methodology facilitates a comprehensive assessment of the antioxidant potential of ethanolic and hexane algae extracts, shedding light on their possible health benefits.

#### 2.7.3. Cytotoxic Activity—Cell Lines

The investigation of the anticancer effects of algae extracts was conducted on human cervical cancer cell lines (HeLa), colon cancer cell lines (HCT-116), and non-transformed human lung fibroblast cell lines (MRC-5). All cell lines used in the experiments were purchased from the American Type Culture Collection (ATCC).

All manipulations were carried out in a class IIa biosafety cabinet under aseptic conditions. HeLa-, HCT-116-, and MRC-5-adherent cell lines were cultured as monolayers in the complete nutrient medium. A liquid-nitrogen-stored cryovial with one milliliter of cell suspension was thawed. The content was transferred to a tube containing 9 mL of complete Dulbecco’s modified Eagle’s medium (DMEM) (Sigma Aldrich, D5671) enriched with 10% fetal bovine serum, L-glutamine (2 mM), non-essential amino acids (0.1 mM) (Sigma Aldrich, M7145), penicillin (100 IU/mL), and streptomycin (100 μg/mL). The tube was then centrifuged for 5 min at 450× *g*. The pelleted cells were resuspended in 5 mL of complete medium and then transferred to a T-25 flask after decanting the supernatant. Cultures were maintained under standard culture conditions of 37 °C and a humidified atmosphere with 5% CO_2_. After reaching sub-confluency, the cells were passaged and transferred to new flasks. Once the flask was emptied of complete DMEM and the cells were washed with PBS, 0.5 mL of a solution containing 0.25% trypsin and 0.05 mM ethylenediaminetetraacetic acid (EDTA) was added to detach the layer of cells. After two min in the incubator under standard conditions, 2.5 mL of supplemented DMEM was added to neutralize the trypsin. One mL of this suspension was transferred to three new T-25 flasks, each filled to a total volume of 5 mL with complete DMEM medium, and then placed back into the incubator under standard conditions.

#### 2.7.4. MTT Assay

The in vitro cytotoxicity effect of seaweed extracts (ethanol and hexane) was evaluated using MTT (3-(4,5-dimethylthiazol-2-yl)-2,5-diphenyltetrazolium bromide) colorimetric assay [41] according to the manufacturer’s instructions. Cells were seeded in 96-well plates at a density of 3 × 10^3^ per well in all experiments. After overnight adherence, the medium was replaced with either a single high concentration of algae (300 µg/mL) or with selected algae with the best cytotoxic profile in a seven-concentration range of 0.3 µg/mL, 1 µg/mL, 3 µg/mL, 10 µg/mL, 30 µg/mL, 100 µg/mL, and 300 µg/mL. Control wells were maintained in supplemented DMEM alone. Following 0, 24, and 48 h incubation, treatment was discarded and in each well was added 100 µL of MTT solution in the final concentration of 0.5 mg/mL. A two-to-four-hour incubation was followed with the dissolution of produced formazan with 150 µL of DMSO (SigmaChemical, St Louis, MO, USA). Optical density was measured with a multi-plate reader (BioTek Epoch Microplate Spectrophotometer, Agilent) at a wavelength of 540 nm. The experiment was repeated in triplicate and three independent experiments were conducted. The percentage of cytotoxicity was calculated using the following equation:

(3) $$\%CT=\frac{A_{control}-A_{treatment}}{A_{control}}\times 100$$

where %CT means percentage of cytotoxicity and A represents absorbance under treatment or control conditions. Cytotoxicity was presented as the mean value from three separate experiments ± standard deviation. The growth inhibition parameters 50% growth inhibition (GI_50_) and total growth inhibition (TGI) were calculated using the National Cancer Institute NCI-60 Screening Methodology recommendations. These parameters, along with 50% inhibitory concentration (IC_50_), were determined using GraphPad Prism 8.0 software. The selectivity index (SI) was calculated as the ratio of the IC_50_ value of MRC-5 to the corresponding IC_50_ value of HeLa and HCT-116.

#### 2.7.5. Annexin V-FITC/7-AAD Assay

The type of cell death induced by seaweed extracts (ethanol and hexane) was identified using the Annexin V-FITC/7-AAD kit (IM364, Beckman Coulter, Brea, CA, USA) according to the manufacturer’s instructions. HeLa and HCT-116 cell lines were seeded at a density of 2 × 10^5^ cells per well in 24-well microtiter plates. Following a 48 h treatment with algae at the corresponding IC_50_ concentration, the cells were trypsinized and washed with PBS. The cells were then resuspended in 100 µL of ice-cold binding buffer (1×), stained with 10 µL Annexin V-FITC (fluorescein 5-isothiocyanate) (and 20 µL 7-AAD (7-amino actinomycin D) and incubated in the dark at room temperature for 15 min. Finally, each tube was filled with 400 µL of binding buffer (1×). Ten thousand cells per sample were analyzed on a Cytomics FC500 flow cytometer (Beckman Coulter, Brea, CA, USA). Data were processed in FlowJo V10 software. Results are presented as representative dot plots and stacked diagrams with mean values ± SD from three separate experiments.

#### 2.7.6. Cell Cycle Analysis

The fluorescent dye propidium iodide (PI) was used to analyze cell cycle perturbation under seaweed extract (ethanol and hexane) treatment of HeLa and HCT-116. Briefly, cells (2 × 10^5^ cells/well) treated with selected algae extracts IC_50_ for 48 h were harvested, washed in PBS, and centrifuged at 450× *g* for 10 min. The cell pellet was then resuspended in 1 mL of cold 70% ethanol. After being incubated overnight at +4 °C, the cells were centrifuged again for five min at 450× *g*. The supernatant was removed, and 1 mL of 500 μg/mL RNase A in PBS was added. Following a 30 min incubation at 37 °C, 5 μL of 10 mg/mL PI was added to the samples, and these were incubated for an additional 15 min in the dark. The final step involved analyzing the 10,000 cells per sample on Cytomics FC500 flow cytometer (Beckman Coulter, Brea, CA, USA). FlowJo V10 software was used to analyze the obtained data.

### 2.8. Statistical Analysis

All analyses were performed in triplicate. The primary factor analyzed is the species of seaweed, with the mean values presented alongside the corresponding standard deviation (SD) values. Statistical analysis was performed using one-way analysis of variance (ANOVA) followed by Tukey’s test with a significance level of *p* ≤ 0.05. Statistical analysis was performed using Statistica v13.3 (Dell Software Inc., Round Rock, TX, USA).

## 3. Results

### 3.1. Protein and Amino Acid Contents

**Figure 1:**
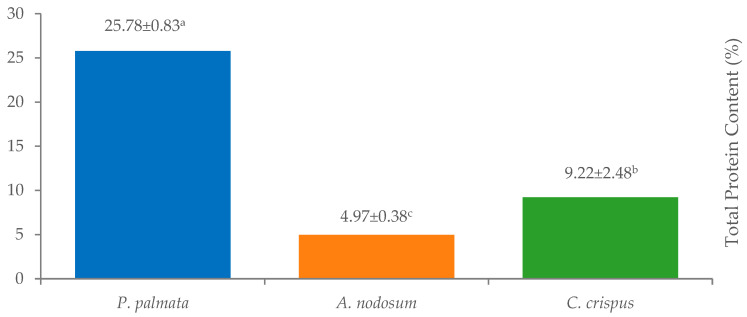
Total protein content (%). Values marked with the same lowercase letters in columns do not differ significantly *p* > 0.05.

The protein content in the seaweed samples varied significantly, ranging from 4.97 ± 0.38% to 25.78 ± 0.83% (Figure 1). *A. nodosum* exhibited the lowest protein percentage at 4.97 ± 0.38%, while *P. palmata* had the highest at 25.78 ± 0.83%. Additionally, *C. crispus* contained a protein content of 9.22 ± 2.48%.

The non-essential amino acid composition of the studied seaweed samples revealed significant variations (Table 1). *C. crispus* had the highest alanine content at 28.79 ± 0.31 g/16 g N, while *P. palmata* and *A. nodosum* had significantly lower levels at 4.21 ± 0.71 g/16 g N and 3.43 ± 0.38 g/16 g N, respectively. Similarly, *C. crispus* exhibited the highest arginine content at 21.47 ± 0.73 g/16 g N, compared with *P. palmata* at 7.67 ± 0.94 g/16 g N and *A. nodosum* at 2.37 ± 0.85 g/16 g N. For aspartic acid, *C. crispus* again showed the highest level at 37.40 ± 0.56 g/16 g N, whereas *P. palmata* and *A. nodosum* had 6.96 ± 1.16 g/16 g N and 6.57 ± 0.66 g/16 g N, respectively. Cysteine was not detected in *P. palmata* but was present in *A. nodosum* and *C. crispus* at 1.85 ± 0.03 g/16 g N and 2.63 ± 0.01 g/16 g N, respectively. The glutamic acid content was highest in *C. crispus* at 51.75 ± 3.03 g/16 g N, while *P. palmata* and *A. nodosum* had 9.56 ± 1.62 g/16 g N and 9.74 ± 0.89 g/16 g N. For glycine, *C. crispus* had the highest level at 17.38 ± 0.56 g/16 g N, followed by *P. palmata* at 2.82 ± 0.42 g/16 g N and *A. nodosum* at 2.51 ± 0.44 g/16 g N. Proline content was also highest in *C. crispus* at 18.13 ± 0.20 g/16 g N, with *P. palmata* and *A. nodosum* having 2.52 ± 0.45 g/16 g N and 1.90 ± 0.21 g/16 g N, respectively. Serine levels were highest in *C. crispus* at 15.93 ± 0.75 g/16 g N, while *P. palmata* and *A. nodosum* had similar amounts at 3.37 ± 0.60 g/16 g N and 3.36 ± 0.68 g/16 g N, respectively. Lastly, tyrosine content was highest in *C. crispus* at 3.78 ± 0.35 g/16 g N, compared with *A. nodosum* at 1.50 ± 1.30 g/16 g N and *P. palmata* at 0.82 ± 0.01 g/16 g N. *C. crispus* consistently demonstrated the highest levels of amino acids compared with *P. palmata* and *A. nodosum*. This indicates that *C. crispus* may be a superior source of non-essential amino acids.

In our study, the essential amino acid profiles of seaweed samples were investigated, and *C. crispus* was found to be particularly rich in these key nutrients compared with *P. palmata* and *A. nodosum* (Table 2). *C. crispus* had the highest isoleucine content at 11.32 ± 0.24 g/16 g N, while *P. palmata* and *A. nodosum* had considerably lower levels at 2.34 ± 0.25 g/16 g N and 1.55 ± 0.19 g/16 g N, respectively. Leucine content was also highest in *C. crispus* at 28.16 ± 0.70 g/16 g N, compared with *P. palmata* at 4.26 ± 0.51 g/16 g N and *A. nodosum* at 3.10 ± 0.12 g/16 g N. Lysine levels were highest in *C. crispus* at 17.28 ± 0.31 g/16 g N, whereas *P. palmata* and *A. nodosum* had 2.57 ± 0.15 g/16 g N and 1.86 ± 0.04 g/16 g N, respectively. For methionine, *C. crispus* had 0.73 ± 0.07 g/16 g N, with *P. palmata* at 0.60 ± 0.09 g/16 g N and *A. nodosum* at 0.85 ± 0.16 g/16 g N. The phenylalanine content was highest in *C. crispus* at 16.88 ± 0.50 g/16 g N, while *P. palmata* and *A. nodosum* had 2.57 ± 0.18 g/16 g N and 2.18 ± 0.10 g/16 g N, respectively. Threonine levels were highest in *C. crispus* at 17.46 ± 0.39 g/16 g N, compared with *P. palmata* at 3.08 ± 0.48 g/16 g N and *A. nodosum* at 2.41 ± 0.28 g/16 g N. Valine content was also highest in *C. crispus* at 16.36 ± 0.08 g/16 g N, with *P. palmata* and *A. nodosum* having 2.92 ± 0.30 g/16 g N and 2.03 ± 0.40 g/16 g N. Lastly, histidine content was highest in *C. crispus* at 11.68 ± 0.47 g/16 g N, while *P. palmata* and *A. nodosum* had 1.76 ± 0.04 g/16 g N and 1.19 ± 0.06 g/16 g N.

### 3.2. Minerals and Trace Elements Contain

Our study comprehensively analyzed the mineral and trace element composition of three types of seaweed: *P. palmata*, *A. nodosum*, and *C. crispus*, expressed in micrograms per gram (µg/g). The results reveal significant variations in nutrient content among the different species (Table 3). *C. crispus* emerged as particularly rich in several minerals. It exhibited the highest calcium content at 51,900 ± 4150 µg/g, which was notably higher than *P. palmata* (10,800 ± 860 µg/g) and *A. nodosum* (15,600 ± 1250 µg/g). In terms of magnesium, *P. palmata* and *A. nodosum* showed similar levels (9300.00 ± 740.00 µg/g and 9610.00 ± 770.00 µg/g, respectively), while *C. crispus* contained slightly lower but comparable amounts (9160 ± 730 µg/g). Potassium levels were relatively consistent among the three species, with *P. palmata*, *A. nodosum*, and *C. crispus* exhibiting concentrations of 21,700 ± 1700 µg/g, 20,800 ± 1700 µg/g, and 19,600 ± 1600 µg/g, respectively. However, sodium content varied significantly, with *A. nodosum* containing the highest levels at 37,300.00 ± 3000.00 µg/g, followed by *C. crispus* (19,100 ± 1500 µg/g) and *P. palmata* (10,600 ± 800 µg/g). In terms of trace elements, *P. palmata* had the highest copper content at 64.0 ± 5.1 µg/g, followed by *C. crispus* (54.5 ± 4.4 µg/g) and *A. nodosum* (45.1 ± 3.6 µg/g). *C. crispus* exhibited the highest iron levels at 610 ± 49 µg/g, contrasting with *P. palmata* (182 ± 15 µg/g) and *A. nodosum* (121 ± 10 µg/g). Similarly, *C. crispus* displayed the highest manganese content at 375.00 ± 30.00 µg/g, significantly exceeding *P. palmata* (168 ± 13 µg/g) and *A. nodosum* (65.1 ± 5.2 µg/g). For zinc, *P. palmata* demonstrated the highest concentration at 101.00 ± 8.00 µg/g, followed closely by *C. crispus* (113 ± 90 µg/g) and *A. nodosum* (67.1 ± 5.4 µg/g). Lead levels were highest in *P. palmata* at 120 ± 10 µg/g, followed by *C. crispus* (94.6 ± 7.6 µg/g) and *A. nodosum* (82.1 ± 6.6 µg/g). Overall, these findings highlight *C. crispus* as being particularly enriched in calcium, iron, manganese, and zinc compared with *P. palmata* and *A. nodosum*.

### 3.3. Fatty Acids Composition

The fatty acid compositions of *P. palmata*, *A. nodosum*, and *C. crispus* were analyzed and are expressed as percentages of total fatty acids in Table 4. The results reveal notable differences among the three seaweeds species in terms of their saturated (SFA), mono-unsaturated (MUFA), and polyunsaturated fatty acids (PUFA) content. For *P. palmata*, the predominant fatty acid was palmitic acid (C 16:0), comprising 55.72 ± 0.25% of its total fatty acids. Other significant fatty acids in *P. palmata* included oleic acid (C 18:1 n9) at 8.33 ± 0.02%, and eicosapentaenoic acid (C 20:5) at 11.28 ± 0.02%. The total saturated fatty acids (SFA) content in *P. palmata* was 62.78 ± 0.23%, with mono-unsaturated fatty acids (MUFA) at 13.99 ± 0.17%, and polyunsaturated fatty acids (PUFA) at 23.26 ± 0.37%. *A. nodosum* showed a markedly different fatty acid profile. The most abundant fatty acid was oleic acid (C 18:1 n9), accounting for 43.368 ± 0.37% of its total fatty acids, followed by myristic acid (C 14:0) at 19.39 ± 0.30%. *A. nodosum* also contained a significant amount of linoleic acid (C 18:2) at 8.27 ± 0.02%. The total SFA in *A. nodosum* was 34.11 ± 0.36%, MUFA was 51.90 ± 0.34%, and PUFA was 13.99 ± 0.02%. *C. crispus* had unique fatty acid characteristics, with a high content of palmitic acid (C 16:0) at 46.763 ± 0.11%, followed by myristic acid (C 14:0) at 17.459 ± 0.01%. It also contained a notable amount of octanoic acid (C 8:0) at 4.99 ± 0.05%. The total SFA content in *C. crispus* was the highest among the three species at 71.85 ± 0.06%, with MUFA at 24.07 ± 0.19%, and PUFA at 4.08 ± 0.25%. In summary, *C. crispus* exhibited the highest proportion of saturated fatty acids, while *A. nodosum* had the highest mono-unsaturated fatty acids content. *P. palmata* was characterized by a balanced distribution of SFAs, MUFAs, and PUFAs, with a significant PUFA fraction. These differences highlight the diverse lipid profiles of the seaweed.

### 3.4. Polyphenols Profile Composition

The polyphenol profile composition of *P. palmata*, *A. nodosum*, and *C. crispus* is presented in Table 5, expressed in micrograms per gram (μg/g). The results highlight the presence and concentrations of various polyphenolic compounds in the three seaweed species. For *P. palmata*, the only detected polyphenol was p-hydroxybenzoic acid, with a concentration of 1.85 ± 0.09 μg/g. *A. nodosum* contained several polyphenols, including kaempferol at 0.79 ± 0.021 μg/g, p-coumaric acid at 0.21 ± 0.024 μg/g, gallic acid at 0.17 ± 0.03 μg/g, and p-hydroxybenzoic acid at 1.86 ± 0.06 μg/g. *C. crispus* showed the presence of chlorogenic acid at 0.41 ± 0.07 μg/g, gallic acid at 0.65 ± 0.02 μg/g, and p-hydroxybenzoic acid at 1.83 ± 0.07 μg/g. The polyphenol profiles of *P. palmata*, *A. nodosum*, and *C. crispus* reveal notable differences in their composition. *P. palmata* was found to have a simpler polyphenol profile, with only p-hydroxybenzoic acid being detected. In contrast, *A. nodosum* exhibited a more diverse polyphenol content, including kaempferol, p-coumaric acid, gallic acid, and p-hydroxybenzoic acid. This indicates a richer array of polyphenolic compounds compared with *P. palmata*. *C. crispus* also demonstrated a diverse polyphenol profile, with the presence of chlorogenic acid, gallic acid, and p-hydroxybenzoic acid. The concentration of p-hydroxybenzoic acid was similar across all three species.

### 3.5. Total Carotenoids

The total carotenoid content (TCC) of the three seaweeds species, *P. palmata*, *A. nodosum*, and *C. crispus*, was determined and is presented in Table 5. The results indicate that *A. nodosum* had the highest TCC at 0.133 mg/g, followed by *P. palmata* with a TCC of 0.084 mg/g, and *C. crispus* with the lowest TCC at 0.050 mg/g. The significant difference in the TCC among these seaweed highlights the varying capacities of these species to accumulate carotenoids. *A. nodosum*, with the highest TCC, may offer more potent antioxidant benefits compared with *P. palmata* and *C. crispus*, which had lower carotenoid levels.

### 3.6. Antioxidant Activity

#### 3.6.1. DPPH Assay

The antioxidant activity of seaweed was evaluated using the DPPH^•^ assay, with Trolox serving as the standard. The IC_50_ value for Trolox was determined to be 2.97 μg/mL, providing a benchmark for the comparison of antioxidant activities among the seaweed samples. The DPPH^•^ assay results for the seaweed are presented in Table 6. The IC_50_ values, indicating the concentration required to inhibit 50% of the DPPH^•^ radical activity, and the Trolox equivalent antioxidant capacity (TEAC) equivalents are reported.

For *P. palmata*, the IC_50_ value in the ethanol extract was found to be 8.43 ± 0.10 mg/mL, with a corresponding TEAC equivalent of 0.000352. In contrast, the hexane extract of *P. palmata* exhibited a significantly lower IC_50_ value of 1.47 ± 0.01 mg/mL, indicating higher antioxidant activity, with a TEAC equivalent of 0.002027.

*A. nodosum* demonstrated a remarkably low IC_50_ value of 0.53 ± 0.02 mg/mL in the ethanol extract, which corresponds to a TEAC equivalent of 0.005552, indicating very high antioxidant activity. The hexane extract of *A. nodosum* had a higher IC_50_ value of 1.32 ± 0.09 mg/mL, with a TEAC equivalent of 0.002253, showing that the antioxidant activity is also high but slightly less than the ethanol extract.

*C. crispus* had an IC_50_ value of 0.93 ± 0.05 mg/mL in the ethanol extract, with a TEAC equivalent of 0.003178. The hexane extract, however, showed a much higher IC_50_ value of 6.87 ± 0.44 mg/mL and a lower TEAC equivalent of 0.000432, indicating significantly lower antioxidant activity compared with the ethanol extract.

When comparing the antioxidant activities between the ethanol and hexane extracts, it is evident that the ethanol extracts generally showed higher antioxidant activity, as indicated by the lower IC_50_ values and higher TEAC equivalents. The ethanol extract of *A. nodosum* exhibited the highest antioxidant activity, with an IC_50_ value of 0.53 ± 0.02 mg/mL. On the other hand, the hexane extract of *C. crispus* demonstrated the lowest antioxidant activity, with an IC_50_ value of 6.87 ± 0.44 mg/mL. This suggests that the choice of solvent significantly affects the measured antioxidant activity, with ethanol generally being the more effective solvent for extracting antioxidant compounds from these seaweeds.

#### 3.6.2. ABTS^+^ Assay

The ABTS^+^ assay was used to evaluate the antioxidant activity of seaweed extracts using Trolox as the standard reference compound, with an IC_50_ value of 2.48 μg/mL. Table 7 presents the results.

For *P. palmata*, the IC_50_ value in the ethanol extract was found to be 0.49 ± 0.01 mg/mL, with a corresponding TEAC equivalent of 0.005039. The hexane extract of *P. palmata* had an IC_50_ value of 0.50 ± 0.01 mg/mL and a TEAC equivalent of 0.005002. These values are very close, indicating similar antioxidant activities for both extracts.

*A. nodosum* showed a significantly lower IC_50_ value of 0.03 ± 0.003 mg/mL in the ethanol extract, with a TEAC equivalent of 0.08078722, indicating very high antioxidant activity. The hexane extract of *A. nodosum* had a higher IC_50_ value of 0.35 ± 0.01 mg/mL, with a TEAC equivalent of 0.00713897, showing that the antioxidant activity is high but less than the ethanol extract.

*C. crispus* had an IC_50_ value of 0.04 ± 0.0007 mg/mL in the ethanol extract, with a TEAC equivalent of 0.0554347. The hexane extract exhibited a much higher IC_50_ value of 1.12 ± 0.02 mg/mL and a lower TEAC equivalent of 0.00222261, indicating significantly lower antioxidant activity when compared with the ethanol extract.

When comparing the antioxidant activities between the ethanol and hexane extracts, the ethanol extracts generally exhibited higher antioxidant activity, as indicated by the lower IC_50_ values and higher TEAC equivalents. The ethanol extract of *A. nodosum* demonstrated the highest antioxidant activity with an IC_50_ value of 0.03 ± 0.003 mg/mL. On the other hand, the hexane extract of *C. crispus* showed the lowest antioxidant activity with an IC_50_ value of 1.12 ± 0.02 mg/mL. This indicates that the choice of solvent significantly impacts the measured antioxidant activity, with ethanol typically being the more effective solvent for extracting antioxidant compounds from these seaweeds.

### 3.7. Anticancer Activity

#### 3.7.1. Cytotoxic Activity of Seaweed Extracts

The cytotoxic effects of the ethanolic (E) and hexane (H) extracts of *P. palmata*, *A. nodosum* and *C. crispus* on human non-transformed fibroblasts (MRC-5), cervical (HeLa) and colorectal (HCT-116) cancer cells were evaluated. The extent of the cytotoxic effect was determined by MTT assay.

**Figure 2:**
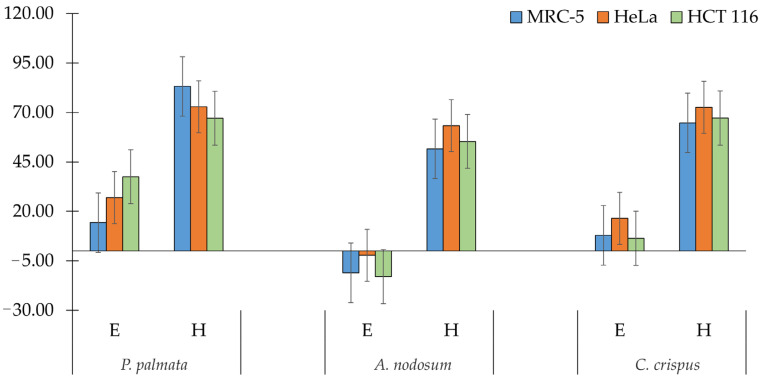
Cytotoxicity of ethanolic (E) and hexane (H) extracts of *P. palmata*, *A. nodosum*, and *C. crispus* (300 μg/mL) on HeLa, HCT-116, and MRC-5 cell lines after 48 h of treatment. Error bars represent the standard deviation (SD) of the mean values from triplicate experiments.

The evaluation was conducted in two stages. In the first stage, screening, cells were treated with a single high concentration (300 µg/mL) of algae extracts for 48 h (Figure 2). Ethanolic extracts of *P. palmata*, *A. nodosum*, and *C. crispus* exhibited minimal to no cytotoxic effect on HeLa and HCT-116 cancer cells. Conversely, hexane extracts of the examined seaweed induced significant cytotoxicity on HeLa and HCT-116, and to a lesser extent on MRC-5. Notably, the *P. palmata* hexane extract demonstrated a very high cytotoxic effect (over 80%) on MRC-5 cells. Therefore, we selected the hexane extract of *A. nodosum* and *C. crispus* for further experiments.

**Figure 3:**
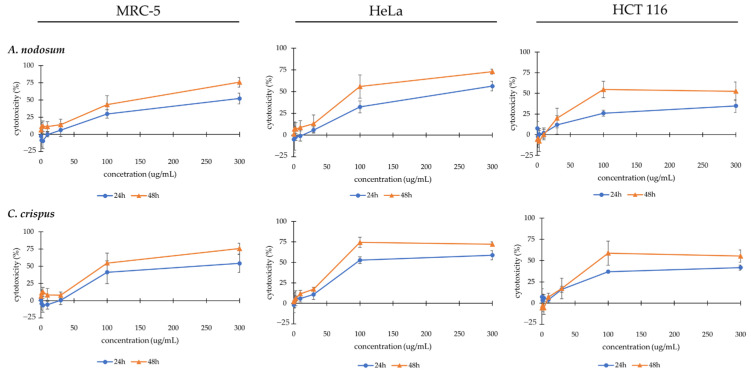
Concentration–response curves for MRC-5, HeLa, and HCT-116 cells treated with hexane extracts of *A. nodosum* and *C. crispus* after 24 and 48 h. The results are presented as mean ± SD.

In the second stage, cells were treated with various concentrations (0.3 μg/mL, 1 μg/mL, 3 μg/mL, 10 μg/mL, 30 μg/mL, 100 μg/mL, and 300 μg/mL) of the hexane extract of *A. nodosum* and *C. crispus* for 24 and 48 h. Results are presented as concentration–response curves (Figure 3) and parameters of the cytotoxic effect (Table 8).

In Figure 3 concentration–response curves are represented. Statistically significant levels of cytotoxicity were detected in all treated cell lines with *A. nodosum* and *C. crispus* hexane extracts increasing with both treatment concentration (30 μg/mL, 100 μg/mL and 300 μg/mL, *p* < 0.05) and exposure time (24 h vs. 48 h, *p* < 0.05). As seen in Table 8, after 48 h of treatment, the *A. nodosum* hexane extract exhibited the lowest IC_50_ value on colorectal carcinoma, indicating statistically significant higher specificity for HCT-116 cells compared with HeLa, which was not observed with the *C. crispus* extract. The *A. nodosum* hexane extract also demonstrated moderate selectivity for HCT-116 cells over non-transformed MRC-5 cells, with an SI value of 2.6. Conversely, selectivity was low towards HeLa cells. The *C. crispus* hexane extract exerted low selectivity toward both cancer cell lines. Cell proliferation of HeLa and HCT-116 cells was significantly halted by treatment of *A. nodosum* and *C. crispus* hexane extracts (*p* < 0.05), respectively, with the lowest TGI values. 

#### 3.7.2. *A. nodosum* and *C. crispus* Induce Apoptosis

**Figure 4:**
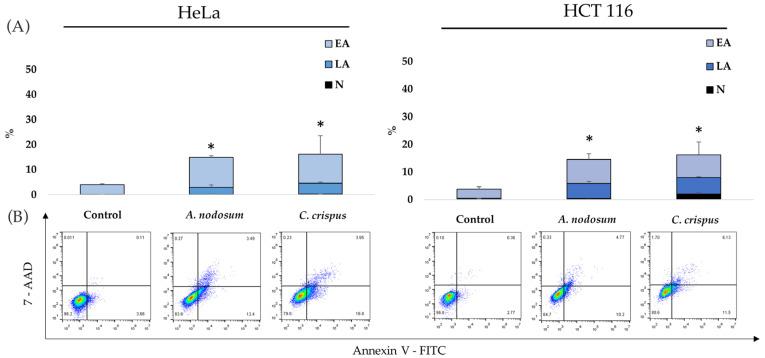
Type of cell death induced by *A. nodosum* and *C. crispus* hexane extracts with the IC_50_ concentration after 48 h. (**A**) Percentage of early (EA), late apoptotic (LA), and necrotic cells (N) in untreated (control) and treated HeLa and HCT-116 cells. Results are presented as mean ± SD for three independent experiments. (**B**) Representative dot plots for HeLa and HCT-116 cells. *—*p* < 0.05.

After exposing cancer cells to the hexane extracts of *A. nodosum* and *C. crispus* with their respective IC_50_ concentrations for 48 h, cell death type was examined using Annexin V/7-AAD. Findings are displayed in Figure 4, showing an early (EA, Annexin+/7-AAD−) and late (LA, Annexin+/7-AAD+) apoptotic, and necrotic (N, Annexin−/7-AAD+) population of cells. Following treatment, the increase in the percentage of apoptotic cells (EA + LA) was low on both cell lines when compared with untreated controls (*p* < 0.05), though it was statistically significant. The population of EA cells was statistically significantly higher in HeLa and HCT-116 cells under treatment with both extracts, followed by LA cells, compared with the control. Very few necrotic cells were present in both cell lines after treatment.

#### 3.7.3. *A. nodosum* and *C. crispus* Induce Cytostatic Effect

**Figure 5:**
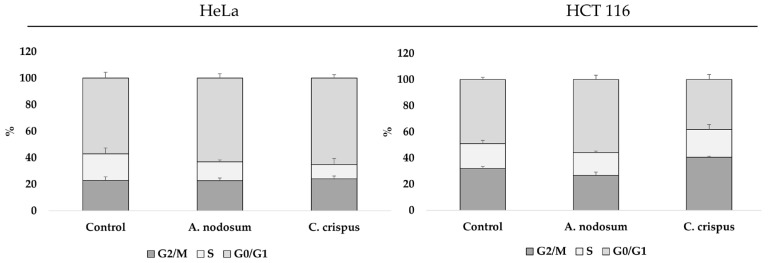
Relative distribution of HeLa and HCT-116 cell cycle phases post-treatment with *A. nodosum* and *C. crispus* hexane extracts with the IC_50_ concentration after 48 h. Results are presented as mean ± SD from three independent experiments.

PI staining in flow cytometry analysis was employed to assess the effect of *A. nodosum* and *C. crispus* hexane extract on the cell cycle progression of HeLa and HCT-116 cells following a 48 h treatment with the IC_50_ concentration. The analysis showed that tested extracts induced perturbances in the cell cycle in both HeLa and HC T116 cells (Figure 5). In the HeLa cell line, both extracts induced cell cycle arrest in the G0/G1 phase, without statistical significance. In the HCT-116 cell line, *A. nodosum* hexane extract arrested cells in the G0/G1 phase (*p* > 0.05), whereas *C. crispus* led to cell accumulation in the G2/M phase (*p* < 0.05).

## 4. Discussion

Our study complements existing research on seaweed protein content, showing variability across different seaweed types. Brown algae (Phaeophyceae) typically have lower protein content (3–15% DW) compared with higher protein contents in green (9–26% DW) and red algae (20–47% DW). These findings underscore the potential of seaweed as protein-rich alternatives in sustainable nutrition strategies and food industries [42]. Our results corroborate previous research, which shows that red algae generally have higher protein content compared with green and brown species [43]. The substantial protein content found in red seaweed species, such as *P. palmata* and *C. crispus*, aligns with existing literature, affirming their potential as significant sources of sustainable protein. This highlights the nutritional richness of seaweed and their versatile applications across various industries, including food and feed [44]. The protein content of green seaweeds, such as those from the *Ulva* genus (Chlorophyta), ranges between 9% and 26% of dry mass, influenced by harvest location and season. Specifically, *Ulva lactuca* and *Ulva rigida* have been reported to contain around 32% protein, with higher values in August and lower in April. Green seaweeds are also rich in essential amino acids, including aspartic acid, glutamic acid, alanine, histidine, arginine, leucine, and threonine [19,45]. In comparison, our study found that red seaweeds, specifically *P. palmata* and *C. crispus*, have significant protein content, aligning with previous reports of higher protein levels in red species. Although we did not specifically measure the protein content in our samples, the literature supports the observation that red seaweed (Rhodophyta) can contain between 20% and 47% protein by dry weight, making them comparable to high-protein vegetables and cereals. This indicates that both green and red seaweed can serve as valuable sources of protein, with their nutritional profiles being influenced by seasonal and environmental factors. The presence of essential amino acids in these seaweeds further enhances their potential as sustainable and nutritious alternatives to traditional protein sources.

Seaweeds are renowned for their adaptation to diverse marine environments, evolving complex metabolic pathways that contribute to their resilience and competitiveness in these habitats. Our analysis of *P. palmata*, *A. nodosum*, and *C. crispus* highlights their varying profiles of non-essential amino acids, with *C. crispus* particularly rich in alanine and glutamic acid. This aligns with the broader exploration of seaweed as a source of diverse bioactive compounds, including proteins and amino acids. The study conducted on *Ulva lactuca*, *Ulva compressa* (formerly *Enteromorpha compressa*) (Chlorophyta), *Padina pavonica* (Phaeophyceae), and *Laurencia obtusa* (Rhodophyta) from Aqaba in 1995 [46] has reported high levels of essential amino acids in all species except for methionine and cystine in *L. obtusa*, and proline in *P. pavonica* and *U. lactuca*. This aligns with our findings, where we observed significant variability in non-essential amino acid profiles among seaweed species like *P. palmata*, *A. nodosum*, and *C. crispus*. Both studies underscore the diverse amino acid compositions of seaweed, highlighting their potential as valuable sources of bioactive compounds and nutrients. When comparing the amino acid composition of seaweeds, it is observed that *Porphyra* species (Rhodophyta), especially *Porphyra dioica* and *Porphyra umbilicalis*, show significantly higher total amino acid content when compared with *Gracilaria vermiculophylla* and *Ulva rigida* species. In addition, red seaweeds, such as *Porphyra* and *Gracilaria vermiculophylla*, show richer essential amino acid profiles such as leucine, valine and threonine [47]. These differences highlight the potential nutritional diversity among seaweed species.

In comparing our study’s findings with the research on seaweeds from the Bulgarian Black Sea coast [11], several key differences and similarities in mineral compositions emerge. Our study focused on seaweed species, including *P. palmata*, *A. nodosum*, and *C. crispus*, revealing significant concentrations of minerals such as calcium, magnesium, potassium, and sodium. In contrast, the Bulgarian study highlighted a broader range of seaweed species with varying mineral contents. For instance, sodium levels ranged from 2.59 to 5.90 mg/g dry weight (DW), potassium from 0.28 to 10.9 mg/g DW, calcium from 5.52 to 21.4 mg/g DW, and magnesium from 2.31 to 4.22 mg/g DW. Iron concentrations varied widely from 6.1 to 105 µg/g DW, zinc from 1.30 to 3.80 µg/g DW, and manganese from 1.60 to 29.4 µg/g DW. The Bulgarian seaweeds generally exhibited lower concentrations of major minerals like sodium, potassium, calcium, and magnesium, when compared with our findings. Ruperez’s study [48] investigated a range of brown and red marine algae, emphasizing their high ash content (21.1% to 39.3%) and sulfate levels (1.3% to 5.9%). Brown algae generally exhibited higher ash content (30.1% to 39.3%) compared with red algae (20.6% to 21.1%). Through atomic absorption spectrophotometry, significant concentrations of macrominerals, such as Na, K, Ca, and Mg, along with trace elements, including Fe, Zn, Mn, and Cu, were identified. Mineral analysis using inductively coupled plasma atomic emission spectroscopy (ICP-OES) demonstrated that both green (*Ulva rigida*, *Codium tomentosum*) and red (*Palmaria palmata*, *Porphyra purpurea*) seaweeds are rich sources of potassium (K) and magnesium (Mg), each containing over 15 g/kg of these minerals. *Ulva rigida* stood out in particular for its significant iron (Fe) content, exceeding 1 g/kg. Additionally, minor concentrations of calcium (Ca), phosphorus (P), and fluorine (F) were detected in these seaweeds [49].

In the study conducted by Mouritsen et al. [50], the total lipid content of the red seaweed *P. palmata* (dulse) ranged from 0.4% to 1.8%. This variation in lipid content was influenced by the location where the seaweed was harvested and the age of the samples. The fatty acid compositions also showed significant diversity, particularly in the levels of polyunsaturated fatty acids, with eicosapentaenoic acid (EPA) being noteworthy, as in our study. The study by Lorenzo et al. [51] investigated the proximate composition and nutritional value of three seaweed species: *Ascophyllum nodosum*, *Fucus vesiculosus*, and *Bifurcaria bifurcate* (Phaeophyceae), with a particular focus on their fatty acid profiles among other nutritional components. The analysis of fatty acid composition across these seaweeds indicated that polyunsaturated fatty acids were the predominant type, followed by saturated fatty acids and mono-unsaturated fatty acids. This finding underscores the nutritional significance of these seaweeds, suggesting that they are rich sources of essential fatty acids beneficial for human health. The study by Koch et al. [52] focused on two red algae species, *Mastocarpus stellatus* and *Chondrus crispus*, which inhabit North Atlantic rocky shores. The study investigated the responses of the algae to high–light stress over a year-long period, specifically during October 2011 and March, May, and August 2012. *Chondrus crispus* was found to be more susceptible to high–light stress, particularly evident during warmer months such as October 2011 and August 2012. In contrast, *Mastocarpus stellatus* demonstrated species-specific adaptations that contribute to its resilience in the upper intertidal zone. These adaptations included higher levels of antioxidants and total lipids, as well as a distinctive fatty acid profile characterized by a specific ratio of shorter-chain to longer-chain fatty acids (C14 + C16/C18 + C20). These characteristics likely enhance *Mastocarpus stellatus*’s ability to withstand high–light and other environmental stresses associated with its habitat variability. These differences underscore the nutritional richness and potential health benefits of these seaweeds, aligning with previous studies that highlight their diverse fatty acid compositions and nutritional significance.

Our study on *P. palmata*, *A. nodosum*, and *C. crispus* revealed varied polyphenol profiles, with *P. palmata* predominantly containing *p*-hydroxybenzoic acid, while *A. nodosum* and *C. crispus* exhibited more diverse compositions, including kaempferol, gallic acid, and others. This contrasts with a Korean study [53] showing sea lettuce and sea mustard as rich sources of polyphenols, correlating with their antioxidative activities. Similarly, Zhang et al. [54] investigated various algal species from the Atlantic coast of Canada and found a strong correlation between total polyphenol content and antioxidant activity. The study on *Macrocystis pyrifera* and *Lessonia spicata* (Phaeophyceae) revealed significant seasonal variations in total polyphenolic content (TPC). Specifically, *Macrocystis pyrifera* exhibited higher TPC levels during winter when compared with other seasons across its different morphological structures. These seasonal fluctuations indicate that winter is a period of peak polyphenolic accumulation in *Macrocystis pyrifera*, which holds importance for industries involved in alginate extraction and other high-value products derived from brown seaweed [55].

Regarding the total carotenoid content, one of the main factors to influence it is the effect of seasonal and geographical differences on the total yield of the target compounds in the extracts [56]. Our study, as well as previous research, highlight significant variability in the carotenoid content among different seaweed species. Similarly, it is important to note that most carotenoids are sensitive to high temperatures and light exposure, which can cause degradation and/or isomerization. Hence, optimizing the extraction process is crucial to achieve the highest yield and purity of these compounds [57]. The primary carotenoids identified in red, green, and brown seaweed species included zeaxanthin, lutein, *β*-carotene, and violaxanthin. Significant variations were observed in the content and composition of carotenoids among these seaweed types. Zeaxanthin and *β*-carotene were detected in all red, green, and brown seaweed, with concentrations ranging from 3.61 to 21.30 μg/g DW and 2.44 to 10.70 μg/g DW, respectively. Violaxanthin was exclusively found in green seaweed at 8.93 μg/g DW, while lutein was specifically present in red seaweed at levels ranging from 9.57 to 38.60 μg/g DW. Green seaweed exhibited the highest total carotenoid content, measuring 100.89 ± 14.71 μg/g DW [58].

Seaweed is a rich source of bioactive compounds, including polysaccharides, antioxidants, minerals, and essential nutrients such as fatty acids, amino acids, and vitamins, making it a valuable functional ingredient. The composition of these biologically active compounds in seaweeds varies due to environmental growth factors, resulting in different compositions even within the same species across the globe. Despite this variability, all seaweeds possess exceptional antioxidant potential [59]. Antioxidants in seaweeds, including chlorophylls, fucoxanthin, vitamins, and phenolic compounds, help prevent oxidative stress, which can damage DNA, proteins, and nucleic acids, leading to diseases like cancer and diabetes. Studies indicate that seaweeds possess higher antioxidant activity than terrestrial plants [60,61,62]. Yuan et al. [63] conducted a study on *Palmaria palmata* to assess its potential as an antioxidant source. They reported that a 1-butanol soluble fraction of *P. palmata* showed significant radical scavenging activity with EC50 concentrations of 12.5 mg/mL for the DPPH^•^ radical and 29.5 mg/mL for the ABTS^+^ radical. Harnedy et al. [64] found that *Palmaria palmata* protein exhibited the highest antioxidant activity, highlighting its potential as a functional food ingredient for promoting health benefits associated with antioxidant properties. Ref. [65] revealed that extracts from *Ascophyllum nodosum* (AN) and *Bifurcaria bifurcata* (BB) (Phaeophyceae), obtained via ultrasound-assisted extraction with water/ethanol, demonstrated superior antioxidant activity compared with microalgae (*Chlorella vulgaris*—Chlorophyta and *Limnosprira platensis*, formerly *Spirulina platensis*—Cyanobacteria). BB extract had the highest extraction yield (35.85 g/100 g DW) and total phenolic content (5.74 g PGE/100 g DW). It also exhibited the highest antioxidant activity in ORAC, DPPH^•^, and FRAP assays, with values of 556.20, 144.65, and 66.50 µmol TE/g DW, respectively, suggesting that BB and AN are promising sources of phenolic antioxidants for potential human consumption. Alkhalaf’s study [66] demonstrated that the methanolic extract of *Chondrus crispus* exhibited significant antioxidant activity. Specifically, at a concentration of 200 µg/mL, the extract markedly decreased levels of free radicals such as DPPH^•^ and ABTS^+^. Moreover, it showed considerable total antioxidant capacity. These results highlight the extract’s potent antioxidant properties, likely due to its high content of flavonoids, phenols (such as catechin, gallic acid, and p-coumaric acid), and tannins. Extraction of *Chondrus crispus* using ultrasonication resulted in the extraction of a soluble fraction with enhanced antioxidant properties. Specifically, the extract exhibited a high antioxidant capacity, with Trolox equivalents of 182.4 mg TEAC/g along with significant levels of gallic acid (13.4 mg/g). These findings underscore the antioxidant potential of *Chondrus crispus* and highlight its suitability for various biological applications [67]. The findings of studies confirm the potential of seaweeds as valuable sources of antioxidant activity. The study highlights specific seaweeds, such as *A. nodosum*, which demonstrated significant antioxidant capabilities in conventional DPPH^•^ and ABTS^+^ assays. Together, these studies, along with ours, underscore the diverse and potent antioxidant properties of seaweeds, suggesting their promising applications in functional foods and nutraceuticals aimed at promoting health and combating diseases related to oxidative stress.

Though their nutritional worth has long been acknowledged, seaweeds have also gathered significant attention as a potential source of new novel anticancer agents, as evidenced by recent studies [66,68,69,70]. These marine organisms are a valuable source of diverse bioactive compounds and have documented miscellaneous pharmacological properties, such as anti-inflammatory, antioxidant and anticancer activity [3]. In Chinese traditional medicine certain seaweeds are recorded as being cures for malignancies [71]. Moreover, there is evidence that consumption of brown algae and their extracts may favor the lower incidence of certain types of cancer [72]. Recognizing this potential, our study also explored the cytotoxic potential of ethanolic, and hexane extracts of *P. palmata*, *A. nodosum*, and *C. crispus*. Our findings reveal that the hexane extract of *A. nodosum* and exhibited significant cytotoxicity against cervical and colorectal cell lines, HeLa and HCT-116, with lower effect in non-transformed human fibroblasts, MRC-5. Specifically, the *A. nodosum* hexane extract exerted overall biological activity and growth inhibition, as well as selectivity for HCT-116 cells, whereas both, *A. nodosum* and *C. crispus* hindered growth of HeLa and HCT-116 cells. Additionally, treatment with examined extracts induced apoptosis and cell cycle arrest in different phases of cancer cell lines. In line with our findings, the anticancer effects of brown and red seaweed extracts and derived bioactive substances have been widely reported [68]. Aqueous extract of *A. nodosum* induced an antiproliferative effect on mouse melanoma cells B16-F0, inhibited colony formation, and induced DNA fragmentation with consequential apoptosis [68]. The significant cytotoxic activity of the methanolic extract of *Sargassum muticum* (Phaeophyceae) against breast cancer cell lines has been documented by Namvar et al. [69]. Additionally, viability of hepatocellular carcinoma (HepG2) and adenocarcinoma human alveolar (A549) cells has been found to be significantly affected under treatment with methanolic extract of *Chondrus crispus* [66]. The notable specificity of *A. nodosum* hexane extract for HCT-116 cells might be attributed to the presence of specific bioactive compounds. Namely, previous research has identified several bioactive compounds in *A. nodosum*, like fucoidan and fucoxanthin, with prominent anticancer activity against colon cancer cell lines [70]. The induction of apoptosis and the inhibition of proliferation in colorectal cancer cells through interfering signaling pathways, such as the PI3K/Akt and MAPK, has been observed under fucoidan treatment [73]. Therefore, the higher cytotoxicity observed in HCT-116 cells may be due to the differential expression of these pathways in colorectal cancer cells, making them more susceptible to the effects of these compounds.

## 5. Conclusions

The investigation into the nutritional composition and bioactive properties of *Palmaria palmata* (dulse), *Ascophyllum nodosum* (knotted wrack), and *Chondrus crispus* (Irish moss) highlights their potential as valuable sources of nutrition and health-promoting compounds. *P. palmata* emerged with the highest protein content (25.78%) among the three seaweeds, underscoring its nutritional value as a protein-rich dietary supplement. *C. crispus*, on the other hand, stood out for its exceptional mineral content, particularly in terms of calcium (51,900 ± 4150 μg/g), iron (610 ± 49 μg/g), manganese (375 ± 30 μg/g), and zinc (113 ± 90 μg/g), making it a significant source of essential minerals. The amino acid analysis revealed *C. crispus* as notably high in both essential and non-essential amino acids, suggesting its potential as a source of complete protein. In terms of fatty acids, *P. palmata* and *A. nodosum* were found to be rich in polyunsaturated fatty acids (PUFAs) (23.26 ± 0.37%, 13.99 ± 0.02%). *A. nodosum* exhibited the highest total carotenoid content (0.13 m/g) among the species studied, indicating its potential as a source of provitamin A compounds and antioxidants. Polyphenol analysis highlighted the presence of various phenolic compounds across all three seaweeds, including *p*-coumaric acid and gallic acid, which are known for their antioxidant properties. Antioxidant activity, assessed using the DPPH^•^ and ABTS^+^ assays, demonstrated that the *A. nodosum* extracts (both ethanol and hexane) exhibited the strongest antioxidant potential, indicating their capacity to scavenge free radicals and protect cells from oxidative damage. While the cytotoxicity evaluation showed that the *A. nodosum* and *C. crispus* hexane extracts exert promising activity against cervical and colorectal cancer, the preliminary findings suggest that these seaweeds possess bioactive compounds that may have therapeutic potential. Overall, the nutritional diversity and bioactive richness of *P. palmata*, *A. nodosum*, and *C. crispus* underscore their suitability as functional foods or dietary supplements aimed at promoting health and preventing the chronic diseases associated with oxidative stress and nutritional deficiencies. Further research is warranted to explore their full therapeutic potential and optimize their incorporation into human diets for maximum health benefits.

## Figures and Tables

**Table 1:** Content of non-essential amino acids in seaweed expressed in (g/16 g N).

Sample	*P. palmata*	*A. nodosum*	*C. crispus*
Alanine	4.21 ± 0.71 ^b^	3.43 ± 0.38 ^b^	28.79 ± 0.31 ^a^
Arginine	7.67 ± 0.94 ^b^	2.37 ± 0.85 ^c^	21.47 ± 0.73 ^a^
Aspartic acid	6.96 ± 1.16 ^b^	6.57 ± 0.66 ^b^	37.40 ± 0.56 ^a^
Cysteine	ND	1.85 ± 0.03 ^b^	2.63 ± 0.01 ^a^
Glutamic acid	9.56 ± 1.62 ^b^	9.74 ± 0.89 ^b^	51.75 ± 3.03 ^a^
Glycine	2.82 ± 0.42 ^b^	2.51 ± 0.44 ^b^	17.38 ± 0.56 ^a^
Proline	2.52 ± 0.45 ^b^	1.90 ± 0.21 ^b^	18.13 ± 0.20 ^a^
Serine	3.37 ± 0.60 ^b^	3.36 ± 0.68 ^b^	15.93 ± 0.75 ^a^
Tyrosine	0.82 ± 0.01 ^c^	1.50 ± 1.30 ^b^	3.78 ± 0.35 ^a^

Values marked with the same lowercase letters in rows do not differ significantly *p* > 0.05. ND—not detected.

**Table 2:** Content of essential amino acids in seaweed expressed in (g/16 g N).

Sample	*P. palmata*	*A. nodosum*	*C. crispus*
Isoleucine	2.34 ± 0.25 ^b^	1.55 ± 0.19 ^c^	11.32 ± 0.24 ^a^
Leucine	4.26 ± 0.51 ^b^	3.10 ± 0.12 ^c^	28.16 ± 0.70 ^a^
Lysine	2.57 ± 0.15 ^b^	1.86 ± 0.04 ^c^	17.28 ± 0.31 ^a^
Methionine	0.60 ± 0.09 ^c^	0.85 ± 0.16 ^ab^	0.73 ± 0.07 ^bc^
Phenylalanine	2.57 ± 0.18 ^b^	2.18 ± 0.10 ^b^	16.88 ± 0.50 ^a^
Threonine	3.08 ± 0.48 ^b^	2.41 ± 0.28 ^c^	17.46 ± 0.39 ^a^
Valine	2.92 ± 0.30 ^b^	2.03 ± 0.40 ^b^	16.36 ± 0.08 ^a^
Histidine	1.76 ± 0.04 ^b^	1.19 ± 0.06 ^b^	11.68 ± 0.47 ^a^

Values marked with the same lowercase letters in rows do not differ significantly *p* > 0.05.

**Table 3:** Minerals and trace elements contained in seaweed expressed in (µg/g).

Mineral	*P. palmata*	*A. nodosum*	*C. crispus*
Calcium (Ca)	10,800 ± 860 ^b^	15,600 ± 1250 ^b^	51,900 ± 4150 ^a^
Magnesium (Mg)	9300 ± 740 ^a^	9610 ± 770 ^a^	9160 ± 730 ^a^
Potassium (K)	21,700 ± 1700 ^a^	20,800 ± 1700 ^a^	19,600 ± 1600 ^a^
Sodium (Na)	10,600 ± 800 ^c^	37,300 ± 3000 ^a^	19,100 ± 1500 ^b^
Copper (Cu)	64.0 ± 5.1 ^a^	45.1 ± 3.6 ^b^	54.5 ± 4.4 ^ab^
Iron (Fe)	182 ± 15 ^b^	121 ± 10 ^c^	610 ± 49 ^a^
Manganese (Mn)	168 ± 13 ^b^	65.1 ± 5.2 ^c^	375 ± 30 ^a^
Zinc (Zn)	101 ± 8 ^a^	67.1 ± 5.4 ^b^	113 ± 90 ^a^
Lead (Pb)	120 ± 10 ^a^	82.1 ± 6.6 ^b^	94.6 ± 7.6 ^ab^

Values marked with the same lowercase letters in rows do not differ significantly *p* > 0.05.

**Table 4:** Fatty acid composition (share in %).

Sample	*P. palmata*	*A. nodosum*	*C. crispus*
C 8:0	ND	ND	4.99 ± 0.05
C 10:0	ND	ND	ND
C 12:0	0.78 ± 0.03	ND	ND
C 14:0	5.56 ± 0.01 ^c^	19.39 ± 0.30 ^a^	17.459 ± 0.01 ^b^
C 14:1	ND	ND	ND
C 15:0	0.72 ± 0.01	ND	ND
C 15:1	ND	ND	ND
C 16:0	55.72 ± 0.25 ^a^	11.85 ± 0.07 ^c^	46.763 ± 0.11 ^b^
C 16:1	3.80 ± 0.017 ^b^	1.47 ± 0.02 ^c^	5.936 ± 0.01 ^a^
C 17:0	ND	ND	ND
C 17:1	ND	ND	ND
C 18:0	ND	0.47 ± 0.21 ^b^	1.504 ± 0.01 ^a^
C 18:1 n9	8.33 ± 0.02 ^c^	43.368 ± 0.37 ^a^	16.895 ± 0.18 ^b^
C 18:1 n7	1.82 ± 0.01	ND	ND
C 18:2	ND	8.27 ± 0.02 ^a^	2.095 ± 0.09 ^b^
C 18:3 n6	ND	ND	ND
C 18:3 n3	4.49 ± 0.01 ^a^	2.656 ± 0.01 ^b^	1.209 ± 0.04 ^c^
C 18:4	ND	ND	ND
C 20:0	ND	0.78 ± 0.01 ^b^	1.128 ± 0.08 ^a^
C 20:2	ND	ND	ND
C 20:4	7.49 ± 0.01	ND	ND
C 20:5	11.28 ± 0.02 ^a^	3.06 ± 0.01 ^b^	0.78 ± 0.12 ^c^
C 22:0	ND	1.61 ± 0.01	ND
C 22:1 n9	ND	7.07 ± 0.14 ^a^	1.24 ± 0.01 ^b^
C 22:6	ND	ND	ND
Not identified	ND	ND	ND
∑SFA *	62.78 ± 0.23 ^b^	34.11 ± 0.36 ^c^	71.85 ± 0.06 ^a^
∑MUFA *	13.99 ± 0.17 ^c^	51.90 ± 0.34 ^a^	24.07 ± 0.19 ^b^
∑PUFA *	23.26 ± 0.37 ^a^	13.99 ± 0.02 ^b^	4.08 ± 0.25 ^c^

* SFA: saturated fatty acid, MUFA: mono-unsaturated fatty acid, PUFA: polyunsaturated fatty acid. Values marked with the same lowercase letters in rows do not differ significantly *p* > 0.05. ND—not detected.

**Table 5:** Polyphenol profile composition (μg/g) and total carotenoid content (TCC) (m/g).

Sample	*P. palmata*	*A. nodosum*	*C. crispus*
Kaempferol	ND	0.79 ± 0.021	ND
Vitexin	ND	ND	ND
Rutin	ND	ND	ND
*p*-Coumaric acid	ND	0.21 ± 0.024	ND
Catechin	ND	ND	ND
Chlorogenic acid	ND	ND	0.41 ± 0.07
Gallic acid	ND	0.17 ± 0.03 ^b^	0.65 ± 0.02 ^a^
*p*-Hydroxybenzoic acid	1.85 ± 0.09 ^a^	1.86 ± 0.06 ^a^	1.83 ± 0.07 ^a^
TCC	0.084 ^a^	0.133 ^b^	0.050 ^c^

Values marked with the same lowercase letters in rows do not differ significantly *p* > 0.05. ND—not detected.

**Table:** Antioxidant activity by DPPH^•^ assay.

Seaweed	IC_50_ (mg/mL)		TEAC equivalent	
	Ethanol Extract	Hexane Extract	Ethanol Extract	Hexane Extract
*P. palmata*	8.43 ± 0.10 ^a^	1.47 ± 0.01 ^a^	0.000352 ^a^	0.002027 ^a^
*A. nodosum*	0.53 ± 0.02 ^b^	1.32 ± 0.09 ^a^	0.005552 ^b^	0.002253 ^a^
*C. crispus*	0.93 ± 0.05 ^c^	6.87 ± 0.44 ^b^	0.003178 ^c^	0.000432 ^b^

Values marked with the same lowercase letters in rows do not differ significantly *p* > 0.05.

**Table 7:** Antioxidant activity by ABTS^+^ assay.

Seaweed	IC_50_ (mg/mL)		TEAC Equivalent	
	Ethanol Extract	Hexane Extract	Ethanol Extract	Hexane Extract
*P. palmata*	0.49 ± 0.01 ^a^	0.50 ± 0.01 ^a^	0.005039 ^a^	0.005002 ^a^
*A. nodosum*	0.03 ± 0.003 ^b^	0.35 ± 0.01 ^b^	0.08078722 ^b^	0.00713897 ^b^
*C. crispus*	0.04 ± 0.0007 ^b^	1.12 ± 0.02 ^c^	0.0554347 ^c^	0.00222261 ^c^

Values marked with the same lowercase letters in rows do not differ significantly *p* > 0.05.

**Table 8:** Parameters of the cytotoxic effect induced by *A. nodosum* and *C. crispus* hexane extract (µg/mL) on MRC-5, HeLa, and HCT-116 cell lines after 48 h of treatment.

Cell line	IC_50_ ± SD	SI	GI_50_ ± SD	TGI ± SD
*A. nodosum*				
MRC-5	113.84 ± 0.16 ^a^	/	45.33 ± 4.69 ^a^	>300 ^a^
HeLa	91.85 ± 0.25 ^a^	1.23	38.27 ± 15.34 ^a^	138.63 ± 37.50 ^a^
HCT-116	43.79 ± 7.07 ^b^	2.6	30.14 ± 10.04 ^a^	64.70 ± 12.4 ^b^
*C. crispus*				
MRC-5	96.70 ± 1.43 ^a^	/	52.84 ± 4.47 ^a^	125.67 ± 12.71 ^a^
HeLa	76.02 ± 0.58 ^a^	1.27	31.51 ± 3.05 ^a^	61.19 ± 2.02 ^b^
HCT-116	65.73 ± 16.4 ^b^	1.47	34.72 ± 31.31 ^a^	115.49 ± 2.75 ^a^

IC_50_—50% biological activity inhibition, GI_50_—50% growth inhibition, TGI—total growth inhibition, SI—selectivity index, SD—standard deviation; values marked with the same lowercase letters in column do not differ significantly (*p* > 0.05).

## Data Availability

The authors declare that all data are embedded in the manuscript. The datasets generated during and/or analyzed during the current study are available from the corresponding author on reasonable request.

## References

[1] Fleurence J. (1999). Seaweed Proteins: Biochemical, Nutritional Aspects and Potential Uses. Trends Food Sci. Technol..

[2] Syad A.N., Shunmugiah K.P., Kasi P.D. (2013). Seaweeds as Nutritional Supplements: Analysis of Nutritional Profile, Physicochemical Properties and Proximate Composition of *G. acerosa* and *S. wightii*. Biomed. Prev. Nutr..

[3] Shannon E., Abu-Ghannam N. (2019). Seaweeds as Nutraceuticals for Health and Nutrition. Phycologia.

[4] Černá M., Kim S.-K. (2011). Chapter 24—Seaweed Proteins and Amino Acids as Nutraceuticals. Advances in Food and Nutrition Research.

[5] Peng Y., Xie E., Zheng K., Fredimoses M., Yang X., Zhou X., Wang Y., Yang B., Lin X., Liu J. (2012). Nutritional and Chemical Composition and Antiviral Activity of Cultivated Seaweed Sargassum Naozhouense Tseng et Lu. Mar. Drugs.

[6] Pliego-Cortés H., Wijesekara I., Lang M., Bourgougnon N., Bedoux G., Bourgougnon N. (2020). Chapter Nine—Current Knowledge and Challenges in Extraction, Characterization and Bioactivity of Seaweed Protein and Seaweed-Derived Proteins. Advances in Botanical Research.

[7] Fleurence J., Morançais M., Dumay J., Yada R.Y. (2018). 9—Seaweed Proteins. Proteins in Food Processing.

[8] Dawczynski C., Schubert R., Jahreis G. (2007). Amino Acids, Fatty Acids, and Dietary Fibre in Edible Seaweed Products. Food Chem..

[9] Galland-Irmouli A.-V., Fleurence J., Lamghari R., Luçon M., Rouxel C., Barbaroux O., Bronowicki J.-P., Villaume C., Guéant J.-L. (1999). Nutritional Value of Proteins from Edible Seaweed *Palmaria palmata* (Dulse). J. Nutr. Biochem..

[10] Circuncisão A.R., Catarino M.D., Cardoso S.M., Silva A.M.S. (2018). Minerals from Macroalgae Origin: Health Benefits and Risks for Consumers. Mar. Drugs.

[11] Panayotova V., Stancheva M. (2013). Mineral Composition of Marine Macroalgae from the Bulgarian Black Sea Coast. Scr. Sci. Medica.

[12] Pereira H., Barreira L., Figueiredo F., Custódio L., Vizetto-Duarte C., Polo C., Rešek E., Engelen A., Varela J. (2012). Polyunsaturated Fatty Acids of Marine Macroalgae: Potential for Nutritional and Pharmaceutical Applications. Mar. Drugs.

[13] Pereira T., Horta A., Barroso S., Mendes S., Gil M.M. (2021). Study of the Seasonal Variations of the Fatty Acid Profiles of Selected Macroalgae. Molecules.

[14] Healy L.E., Zhu X., Pojić M., Sullivan C., Tiwari U., Curtin J., Tiwari B.K. (2023). Biomolecules from Macroalgae—Nutritional Profile and Bioactives for Novel Food Product Development. Biomolecules.

[15] Santos S.A.O., Félix R., Pais A.C.S., Rocha S.M., Silvestre A.J.D. (2019). The Quest for Phenolic Compounds from Macroalgae: A Review of Extraction and Identification Methodologies. Biomolecules.

[16] Vlaisavljević S., Rašeta M., Berežni S., Passamonti S., Tramer F. (2021). Four Selected Commercial Seaweeds: Biologically Active Compounds, Antioxidant and Cytotoxic Properties. Int. J. Food Sci. Nutr..

[17] El-Rafie H.M., Hammam H.H., Ahmed E.A.-E. (2024). Nutritional Values, Antioxidant, and Cytotoxic Activities of Selected Edible Marine Macroalgae: A Comparative Study. Food Meas..

[18] Subbiah V., Xie C., Dunshea F.R., Barrow C.J., Suleria H.A.R. (2023). The Quest for Phenolic Compounds from Seaweed: Nutrition, Biological Activities and Applications. Food Rev. Int..

[19] Jesumani V., Du H., Aslam M., Pei P., Huang N. (2019). Potential Use of Seaweed Bioactive Compounds in Skincare—A Review. Mar. Drugs.

[20] Ismail M.M., Alotaibi B.S., EL-Sheekh M.M. (2020). Therapeutic Uses of Red Macroalgae. Molecules.

[21] Deepika C., Ravishankar G.A., Rao A.R., Ranga Rao A., Ravishankar G.A. (2022). Potential Products from Macroalgae: An Overview. Sustainable Global Resources of Seaweeds Volume 1: Bioresources, Cultivation, Trade and Multifarious Applications.

[22] Garcia-Oliveira P., Carreira-Casais A., Caleja C., Pereira E., Calhelha R.C., Sokovic M., Simal-Gandara J., Ferreira I.C.F.R., Prieto M.A., Barros L. (2020). Macroalgae as an Alternative Source of Nutrients and Compounds with Bioactive Potential. Proceedings.

[23] Garicano Vilar E., O’Sullivan M.G., Kerry J.P., Kilcawley K.N. (2020). Volatile Compounds of Six Species of Edible Seaweed: A Review. Algal Res..

[24] Stévant P., Schmedes P.S., Le Gall L., Wegeberg S., Dumay J., Rebours C. (2023). Concise Review of the Red Macroalga Dulse, *Palmaria palmata* (L.) Weber & Mohr. J. Appl. Phycol..

[25] MacArtain P., Gill C.I.R., Brooks M., Campbell R., Rowland I.R. (2008). Nutritional Value of Edible Seaweeds. Nutr. Rev..

[26] Obluchinskaya E.D., Pozharitskaya O.N., Gorshenina E.V., Daurtseva A.V., Flisyuk E.V., Generalova Y.E., Terninko I.I., Shikov A.N. (2024). *Ascophyllum nodosum* (Linnaeus) Le Jolis from Arctic: Its Biochemical Composition, Antiradical Potential, and Human Health Risk. Mar. Drugs.

[27] Pereira L., Morrison L., Shukla P.S., Critchley A.T. (2020). A Concise Review of the Brown Macroalga *Ascophyllum nodosum* (Linnaeus) Le Jolis. J. Appl. Phycol..

[28] Collén J., Cornish M.L., Craigie J., Ficko-Blean E., Hervé C., Krueger-Hadfield S.A., Leblanc C., Michel G., Potin P., Tonon T. (2014). Chondrus crispus—A Present and Historical Model Organism for Red Seaweeds. Advances in Botanical Research.

[29] Park S.-J., Sharma A., Lee H.-J. (2024). An Update on the Chemical Constituents and Biological Properties of Selected Species of an Underpinned Genus of Red Algae: Chondrus. Mar. Drugs.

[30] (2013). Cereals and Pulses—Determination of the Nitrogen Content and Calculation of the Crude Protein Content—Kjeldahl Method.

[31] AOAC Official Method 994.12 Amino Acids in Feeds|PDF|Amino Acid|pH. https://www.scribd.com/document/609975831/Aoac-Official-Method-994-12-Amino-Acids-in-Feeds-1.

[32] Tomczak A., Zielińska-Dawidziak M., Piasecka-Kwiatkowska D., Lampart-Szczapa E. (2018). Blue Lupine Seeds Protein Content and Amino Acids Composition. Plant Soil Environ..

[33] Matusiewicz H., Ślachciński M. (2019). A Comparison of ETV and LA for the Determination of Trace Elements in Solid Samples by MIP OES. Ecol. Chem. Eng. S.

[34] Folch J., Lees M., Stanley G.H.S. (1957). A Simple Method for the Isolation and Purification of Total Lipides from Animal Tissues. J. Biol. Chem..

[35] American Oil Chemists’ Society (2005). AOCS Official Method Ce 1h-05: Determination of Cis-, Trans-, Saturated, Monounsaturated and Polyunsaturated Fatty Acids in Vegetable or Non-ruminant Animal Oils and Fats by Capillary GLC. Official Methods and Recommended Practices of the AOCS. https://www.sciepub.com/reference/251862.

[36] Kowalczewski P.Ł., Gumienna M., Rybicka I., Górna B., Sarbak P., Dziedzic K., Kmiecik D. (2021). Nutritional Value and Biological Activity of Gluten-Free Bread Enriched with Cricket Powder. Molecules.

[37] (1986). Determination of Vitamin A and Its Provitamins.

[38] Drożdżyńska A., Dzidzic K., Kośmider A., Leja K., Czaczyk K., Górecka D. (2012). Application of Fast Liquid Chromatography for Antioxidants Analysis. Acta Sci. Pol. Technol. Aliment..

[39] Čmiková N., Kowalczewski P.Ł., Kmiecik D., Tomczak A., Drożdżyńska A., Ślachciński M., Królak J., Kačániová M. (2024). Characterization of Selected Microalgae Species as Potential Sources of Nutrients and Antioxidants. Foods.

[40] Kačániová M., Vukovic N.L., Čmiková N., Galovičová L., Schwarzová M., Šimora V., Kowalczewski P.Ł., Kluz M.I., Puchalski C., Bakay L. (2023). *Salvia sclarea* Essential Oil Chemical Composition and Biological Activities. Int. J. Mol. Sci..

[41] Mosmann T. (1983). Rapid Colorimetric Assay for Cellular Growth and Survival: Application to Proliferation and Cytotoxicity Assays. J. Immunol. Methods.

[42] Gordalina M., Pinheiro H.M., Mateus M., Da Fonseca M.M.R., Cesário M.T. (2021). Macroalgae as Protein Sources—A Review on Protein Bioactivity, Extraction, Purification and Characterization. Appl. Sci..

[43] Harnedy P.A., FitzGerald R.J. (2011). Bioactive Proteins, Peptides, and Amino Acids from Macroalgae1: Macroalgae: Bioactive Agent Source. J. Phycol..

[44] O’Brien R., Hayes M., Sheldrake G., Tiwari B., Walsh P. (2022). Macroalgal Proteins: A Review. Foods.

[45] Ortiz J., Uquiche E., Robert P., Romero N., Quitral V., Llantén C. (2009). Functional and Nutritional Value of the Chilean Seaweeds *Codium fragile*, *Gracilaria chilensis* and *Macrocystis pyrifera*. Eur. J. Lipid Sci. Technol..

[46] Wahbeh M.I. (1997). Amino Acid and Fatty Acid Profiles of Four Species of Macroalgae from Aqaba and Their Suitability for Use in Fish Diets. Aquaculture.

[47] Machado M., Machado S., Pimentel F.B., Freitas V., Alves R.C., Oliveira M.B.P.P. (2020). Amino Acid Profile and Protein Quality Assessment of Macroalgae Produced in an Integrated Multi-Trophic Aquaculture System. Foods.

[48] Ruperez P. (2002). Mineral Content of Edible Marine Seaweeds. Food Chem..

[49] Echave J., Lourenço-Lopes C., Carreira-Casais A., Chamorro F., Fraga-Corral M., Otero P., Garcia-Perez P., Baamonde S., Fernández-Saa F., Cao H. (2021). Nutritional Composition of the Atlantic Seaweeds *Ulva rigida*, *Codium tomentosum*, *Palmaria palmata* and *Porphyra purpurea*. Chem. Proc..

[50] Mouritsen O.G., Dawczynski C., Duelund L., Jahreis G., Vetter W., Schröder M. (2013). On the Human Consumption of the Red Seaweed Dulse (*Palmaria palmata* (L.) Weber & Mohr). J. Appl. Phycol..

[51] Lorenzo J., Agregán R., Munekata P., Franco D., Carballo J., Şahin S., Lacomba R., Barba F. (2017). Proximate Composition and Nutritional Value of Three Macroalgae: *Ascophyllum nodosum*, *Fucus vesiculosus* and *Bifurcaria bifurcata*. Mar. Drugs.

[52] Koch K., Hagen W., Graeve M., Bischof K. (2017). Fatty Acid Compositions Associated with High-Light Tolerance in the Intertidal Rhodophytes *Mastocarpus stellatus* and *Chondrus crispus*. Helgol. Mar. Res..

[53] Kwak C.-S., Kim S.-A., Lee M.-S. (2005). The Correlation of Antioxidative Effects of 5 Korean Common Edible Seaweeds and Total Polyphenol Content. J. Korean Soc. Food Sci. Nutr..

[54] Zhang Q., Zhang J., Shen J., Silva A., Dennis D.A., Barrow C.J. (2006). A Simple 96-Well Microplate Method for Estimation of Total Polyphenol Content in Seaweeds. J. Appl. Phycol..

[55] Beratto-Ramos A., Castillo-Felices R.d.P., Troncoso-Leon N.A., Agurto-Muñoz A., Agurto-Muñoz C. (2019). Selection Criteria for High-Value Biomass: Seasonal and Morphological Variation of Polyphenolic Content and Antioxidant Capacity in Two Brown Macroalgae. J. Appl. Phycol..

[56] Heffernan N., Smyth T.J., FitzGerald R.J., Vila-Soler A., Mendiola J., Ibáñez E., Brunton N.P. (2016). Comparison of Extraction Methods for Selected Carotenoids from Macroalgae and the Assessment of Their Seasonal/Spatial Variation. Innov. Food Sci. Emerg. Technol..

[57] Cikoš A.-M., Šubarić D., Roje M., Babić J., Jerković I., Jokić S. (2022). Recent Advances on Macroalgal Pigments and Their Biological Activities (2016–2021). Algal Res..

[58] Othman R., Amin N.A.M., Bakar A.E.A., Fadzillah N.A., Mahmad N. (2019). Carotenoid Pigments of Red, Green and Brown Macroalgae Species as Potential Active Pharmaceutical Ingredients. J. Pharm. Nutr. Sci..

[59] Kumar Y., Tarafdar A., Badgujar P.C. (2021). Seaweed as a Source of Natural Antioxidants: Therapeutic Activity and Food Applications. J. Food Qual..

[60] Chan P.T., Matanjun P. (2017). Chemical Composition and Physicochemical Properties of Tropical Red Seaweed, *Gracilaria changii*. Food Chem..

[61] Chew Y.L., Lim Y.Y., Omar M., Khoo K.S. (2008). Antioxidant Activity of Three Edible Seaweeds from Two Areas in South East Asia. LWT Food Sci. Technol..

[62] O’Sullivan A.M., O’Callaghan Y.C., O’Grady M.N., Queguineur B., Hanniffy D., Troy D.J., Kerry J.P., O’Brien N.M. (2011). In Vitro and Cellular Antioxidant Activities of Seaweed Extracts Prepared from Five Brown Seaweeds Harvested in Spring from the West Coast of Ireland. Food Chem..

[63] Yuan Y.V., Bone D.E., Carrington M.F. (2005). Antioxidant Activity of Dulse (*Palmaria palmata*) Extract Evaluated In Vitro. Food Chem..

[64] Harnedy P.A., FitzGerald R.J. (2013). In Vitro Assessment of the Cardioprotective, Anti-Diabetic and Antioxidant Potential of *Palmaria palmata* Protein Hydrolysates. J. Appl. Phycol..

[65] Agregán R., Munekata P., Franco D., Carballo J., Barba F., Lorenzo J. (2018). Antioxidant Potential of Extracts Obtained from Macro- (*Ascophyllum nodosum*, *Fucus vesiculosus* and *Bifurcaria bifurcata*) and Micro-Algae (Chlorella Vulgaris and Spirulina Platensis) Assisted by Ultrasound. Medicines.

[66] Alkhalaf M.I. (2021). Chemical Composition, Antioxidant, Anti-Inflammatory and Cytotoxic Effects of *Chondrus crispus* Species of Red Algae Collected from the Red Sea along the Shores of Jeddah City. J. King Saud Univ.-Sci..

[67] Torres M.D., Flórez-Fernández N., Domínguez H. (2021). *Chondrus crispus* Treated with Ultrasound as a Polysaccharides Source with Improved Antitumoral Potential. Carbohydr. Polym..

[68] Lee S.-U., Kim Y.H. (2022). Anti-cancer effects of kelp extract in mouse melanoma B16-F0 cell line through apoptosis. Korean J. Food Sci. Technol..

[69] Namvar F., Mohamad R., Baharara J., Zafar-Balanejad S., Fargahi F., Rahman H.S. (2013). Antioxidant, Antiproliferative, and Antiangiogenesis Effects of Polyphenol-Rich Seaweed (*Sargassum Muticum*). BioMed Res. Int..

[70] Peng J., Yuan J.-P., Wu C.-F., Wang J.-H. (2011). Fucoxanthin, a Marine Carotenoid Present in Brown Seaweeds and Diatoms: Metabolism and Bioactivities Relevant to Human Health. Mar. Drugs.

[71] Maruyama H., Yamamoto I., Bird C.J., Ragan M.A. (1984). An Antitumor Fucoidan Fraction from an Edible Brown Seaweed, Laminaria Religiosa. Proceedings of the Eleventh International Seaweed Symposium.

[72] Fitton J.H. (2003). Brown Marine Algae: A Survey of Therapeutic Potentials. Altern. Complement. Ther..

[73] Atashrazm F., Lowenthal R., Woods G., Holloway A., Dickinson J. (2015). Fucoidan and Cancer: A Multifunctional Molecule with Anti-Tumor Potential. Mar. Drugs.

